# Human Spotted Fever Rickettsial Infections

**DOI:** 10.3201/eid1104.040287

**Published:** 2005-04

**Authors:** George B. Schoeler, Cecilia Morón, Allen Richards, Patrick J. Blair, James G. Olson

**Affiliations:** * U.S. Navy Disease Vector Ecology and Control Center, Silverdale, Washington, USA;; † Ministry of Health, Lima, Peru;; ‡ U.S. Naval Medical Research Center, Silver Spring, Maryland, USA;; § U.S. Naval Medical Research Center Detachment, Lima, Peru

**Keywords:** Rickettsial disease, South America, disease surveillance, spotted fever group *Rickettsia*, dispatch

## Abstract

Serum specimens from patients at 4 sites in Peru were tested for evidence of spotted fever group rickettsial infection. Results showed that 30 (18%) of 170 patients had spotted fever group rickettsial infections, which likely caused their illnesses. These findings document laboratory-confirmed spotted fever from diverse areas of Peru.

Rickettsial spotted fever was first described in South America in 1931 in Sao Paulo, Brazil (*1*). The etiologic agent, *Rickettsia rickettsii*, and the tick vector, *Amblyomma cajenennse* (the Cayenne tick), were subsequently identified. Serologic evidence of *R. rickettsii* infections has been documented in several countries in South and central America, including Argentina (*2*), Brazil and Uruguay (*3*), Colombia (*4*), Costa Rica (*5*), Panama (*6*), and Mexico (*7*). A recent study documented for the first time serologic evidence for spotted fever group (SFG) *Rickettsia* infections in 1 region of northern Peru (*8*). We describe serologic evidence of SFG rickettsial infections in diverse areas of Peru, including laboratory-confirmed infections among patients with clinical febrile disease.

## The Study

Serum samples were obtained from 4 areas in Peru: Chiclayito and Salitral (Piura Department); La Merced (Junin Department); and Cusco (Cusco Department) (Figure). Chiclayito is a small village (population 6,133) ≈30 m above sea level on the outskirts of the city of Piura in the northern coastal desert. Salitral is a small rural village (population 1,503) ≈162 m above sea level in a more temperate region of the Salitral District (Morropon Province, Piura Department) ≈3 h by car from Chiclayito. La Merced is the capital of the Chanchamayo District (Chanchamayo Province, Junín Department) and located ≈751 m above sea level ≈350 km east of the Peruvian capital city of Lima, on the eastern side of the Andes. The district has a population of 31,000; approximately half live in La Merced. Cusco (population 260,000) is located ≈3,350 m above sea level in the southern Peruvian Andes 1,089 km southeast of Lima.

**Figure:**
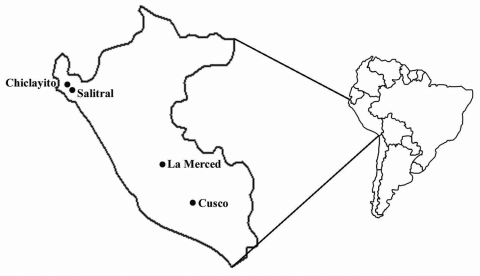
Four study sites in Peru surveyed for human spotted fever rickettsial infections.

Sera from patients representing the 4 surveillance sites were tested for antibodies against SFG rickettsiae after written informed consent was provided by each patient (Department of Defense Institutional Review Board No. 31535). Patients enrolled had a fever ≥38°C and at least 2 other signs or symptoms including headache, myalgia, arthralgia, rash, and bleeding. Patients with a positive blood film for malarial parasites or obvious disease such as diarrhea or upper respiratory illness were excluded.

Paired (acute- and convalescent-phase) patient serum samples were evaluated for immunoglobulin (Ig) G antibodies reactive with *R. rickettsii* antigen by either an indirect immunofluorescence assay (IFA) or enzyme immunoassay (EIA). Serum specimens were also tested by IFA for typhus group rickettsial antibodies and were uniformly negative. IFA analysis was conducted according to directions provided by the manufacturer (PanBio, INDX, Inc., Baltimore, MD, USA). Endpoint titers were recorded as the reciprocal of the last dilution exhibiting specific fluorescence. Titers ≥1:64 were considered positive. Patients with confirmed spotted fever were those who showed a ≥4-fold increase in *R. rickettsii* IgG titer from acute phase to convalescent phase of illness.

The EIA was conducted by using a 4-step indirect immunoassay to detect *R. rickettsii* IgG, as described (*8*). A positive serum dilution exceeded the mean plus 3 standard deviations between the absorbance of *R. rickettsii* antigen and the negative control antigen of 5 control serum specimens. Serum samples were titrated to endpoint and the highest dilution found positive was recoded as the *R. rickettsii* IgG titer. Serum from a serologically confirmed case-patient showed a ≥4-fold increase in antibody titer from the acute to the convalescent phase.

A total of 170 patients, 50 from Chiclayito and the Salitral Health Centers (Piura Department), 67 from Cusco Hospital (Cusco Department), and 53 from La Merced Hospital (Junin Department), were tested for antibodies to SFG rickettsiae. IFA testing was done at the Peruvian National Institute of Health, while EIAs were conducted at Naval Medical Research Center Detachment. Not all patients were tested by both assays (Table 1). Of the 170 patients tested, 30 (18%) yielded results that suggested that SFG rickettsial infections were the most likely cause of their illnesses (Table 1). Patients from all 4 study sites in 3 departments of Peru had evidence of SFG rickettsiae infections as the cause of illness. Frequencies of confirmed patients in the 3 departments did not differ significantly (p > 0.52). Table 2 shows the frequencies of spotted fever by age and sex for the 164 patients for whom data were recorded. Age groups did not differ significantly (p > 0.5). The frequency of spotted fever was 27% in female patients and 10% in male patients (p < 0.005).

**Table 1:** EIA and IFA test results for antibodies to *Rickettsia rickettsii* among patients from 3 departments of Peru*

Department	EIA		IFA		EIA and/or IFA	
	No. tested†	No. positive (%)‡	No. tested†	No. positive (%)§	No. tested†	No. positive (%)
Cusco	36	6 (17)	56	11 (20)	67	16 (24)
Junin	42	8 (19)	19	2 (11)	53	10 (19)
Piura	50	4 (8)	0		50	4 (8)
Total	128	18 (14)	75	13 (17)	170	30 (18)

*By enzyme immunoassay (EIA) or indirect immunofluorescence antibody assay (IFA). †Febrile patients from whom acute- and convalescent-phase serum specimens were available for testing. ‡Criterion for confirmation was a ≥4-fold increase in *R. rickettsii* immunoglobulin (Ig) G antibody titer from acute to convalescent phase of illness by EIA. §Criterion for confirmation was a ≥4-fold increase in *R. rickettsii* IgG antibody titer from acute to convalescent phase of illness by IFA.

**Table 2:** Spotted fever frequency by age and sex

Age (y)	Male patients			Female patients		
	No. positive	No. tested	% positive	No. positive	No. tested	% positive
5–10	2	21	11	3	14	21
11–15	2	16	14	3	12	25
16–20	2	17	13	3	10	30
21–30	1	17	6	7	23	30
>30	2	19	12	4	15	27
Total	9	90	10	20	74	27

The signs and symptoms of patients with confirmed spotted fever who came to the treatment facility included fever and malaise (100%), chills (94%), weakness (94%), shortness of breath (94%), prostration (81%), arthralgia (62%), abdominal pain (62%), cough (56%), nausea (56%), and runny nose (56%). None of the patients died, and most patients had a relatively mild febrile illness. There were no clear clinical differences in patients with confirmed cases of spotted fever compared with febrile patients who did not have spotted fever.

Evidence of SFG rickettsial infection was observed in samples taken from febrile patients in Cusco, Junin, and Piura departments. The etiologic agent or agents responsible for the spotted fever illnesses remain unknown. Appropriate samples from these patients were not available for isolation or molecular identification by a polymerase chain reaction.

## Conclusions

Host inflammation may partly contribute to the pathogenic sequelae with intra-endothelial cell infection in more severe SFG infection (*9*). Patients infected with *R. akari* typically experience a mild and or asymptomatic disease characterized by low-grade fever, sweats, headache, and a vesicular eruption over the trunk and extremities (*10*). *R. akari* is maintained transovarially in the mite vector and transmitted to humans by the house mouse mite (*Liponyssoides sanguineus*). Infections have generally been reported among higher risk populations such as intravenous drug users (*11*), or within the densely populated inner city (*12*). Less is known about the susceptibility of rural agrarian populations. The concentration of humans in close proximity to house mice and their mites are factors that could contribute to an increase in rickettsialpox in the region. Sporadic cases of rickettsialpox may be confused with chickenpox, a common illness associated vesicular rash. However, none of the confirmed SFG rickettsia-infected patients had vesicular rashes typical of rickettsialpox.

Cat flea typhus, caused by *R. felis*, is a mild disease similar to murine typhus (*13*). Typical clinical findings include fever, headache, and occasional rash. The clinical manifestations of patients infected with SFG rickettsiae are similar to those described for cat flea typhus. However, recent discoveries of novel rickettsioses caused by distinct SFG rickettsiae in Europe, Africa, Australia, Asia, and North America during the last 25 years (*14**,**15*) suggest that the infections reported in this study may be the results of a novel SFG rickettsial agent. Future work is needed to identify the agent involved and to clearly link clinical signs and symptoms with diagnoses.

The higher frequency of cases in women suggests occupational exposure since in these areas of Peru women are generally more involved with domestic activities near the home. Possibilities for increased exposure of women may include more frequent work in the fields, thus exposing them to arthropod vectors; closer contact with domestic animals that may be involved in maintaining the SFG rickettsial agent (although no evidence was collected to support this); or exposure to house mouse mites in the home. Serologic evidence suggests that SFG rickettsiae were responsible for causing febrile illnesses in these 4 study sites of Peru, which demonstrates that SFG rickettsia result in human disease in Peru. Further studies are needed to document the species of SFG rickettsiae and to determine the vectors of these rickettsial infections. In addition, epidemiologic studies are needed to identify the risk factors, document the clinical spectrum, and suggest public health recommendations for prevention.
